# Supplementary material for: Polyketide synthase gene clusters in the ericoid mycorrhizal fungus Oidiodendron maius: genome-wide prediction, expression profiling, and functional characterization of knockout mutants
Source: Front Plant Sci. 2026 Sep 16;17:1924430. doi: 10.3389/fpls.2026.1924430 (PMC13623903; doi:10.3389/fpls.2026.1924430)
Supplement: Supplementary file 1 [file SupplementaryFile1.zip › Supplementary Materials/Supplementary Folder 1.zip!/Oidma1_AssemblyScaffolds_Repeatmasked/knownclusterblast/cluster49/jgi.p_Oidma1_136720_mibig_hits.html]

| MIBiG Protein | Description | MIBiG Cluster | MiBiG Product | % ID | % Coverage | BLAST Score | E-value |
| --- | --- | --- | --- | --- | --- | --- | --- |
| CDM36724.1 | Cytochrome\_P450 | BGC0001360 | Mycophenolic\_acid\_biosynthetic\_gene\_cluster | 49 | 96.6336633663 | 504 | 1.6e-142 |
| EED17999.1 | benzoate\_4-monooxygenase\_cytochrome\_P450,\_putative | BGC0000154 | Stipitatic\_acid\_biosynthetic\_gene\_cluster | 50 | 74.4554455446 | 393 | 3.9e-109 |
| ADY00131.1 | putative\_P450\_monooxygenase | BGC0000104 | Mycophenolic\_acid\_biosynthetic\_gene\_cluster | 47 | 68.7128712871 | 323 | 5e-88 |
| EAQ84780.1 | hypothetical\_protein | BGC0001219 | Shanorellin\_biosynthetic\_gene\_cluster | 53 | 43.1683168317 | 252 | 8.3e-67 |
| ADI24930.1 | VrtE | BGC0000168 | Viridicatumtoxin\_biosynthetic\_gene\_cluster | 31 | 102.178217822 | 237 | 4.7e-62 |
| AGC83578.1 | P450\_monooxygenase | BGC0000818 | Notoamide\_biosynthetic\_gene\_cluster | 26 | 89.3069306931 | 144 | 3.2e-34 |
| ADM34140.1 | P450 | BGC0001084 | Notoamide\_/\_stephacidin\_biosynthetic\_gene\_cluster | 26 | 89.504950495 | 139 | 1e-32 |
| AAK33070.1 | trichothecene\_C-15\_hydroxylase | BGC0001278 | Nivalenol\_/\_deoxynivalenol\_/\_3-acetyldeoxynivalenol\_/ | 21 | 95.2475247525 | 114 | 4.6e-25 |
| BAH23996.1 | cytochrome\_P450 | BGC0000356 | Fumitremorgin\_biosynthetic\_gene\_cluster | 22 | 95.4455445545 | 111 | 3.9e-24 |
| AIA58898.1 | putative\_cytochrome\_P450 | BGC0001141 | Brefeldin\_biosynthetic\_gene\_cluster | 24 | 95.6435643564 | 101 | 3.1e-21 |
| EAL89312.1 | cytochrome\_P450\_monooxygenase,\_putative | BGC0000686 | Helvolic\_acid\_biosynthetic\_gene\_cluster | 25 | 96.4356435644 | 99 | 1.2e-20 |
| BAV32152.1 | cytochrome\_P450\_monooxygenase | BGC0001373 | Sordarin\_biosynthetic\_gene\_cluster | 23 | 99.2079207921 | 91 | 2.4e-18 |
| KGN46390.1 | hypothetical\_protein | BGC0001315 | Cucurbitacin\_biosynthetic\_gene\_cluster. | 22 | 88.7128712871 | 89 | 9.3e-18 |
| ABV57820.1 | cytochrome\_P450\_monooxygenase | BGC0001241 | Ergotamine\_biosynthetic\_gene\_cluster | 27 | 35.0495049505 | 84 | 3.9e-16 |
| Bcin12g06400.1 |  | BGC0000631 | Botrydial\_biosynthetic\_gene\_cluster | 22 | 93.6633663366 | 81 | 4.3e-15 |
| AEE83585.1 | cytochrome\_P450,\_family\_705,\_subfamily\_A,\_polypeptide\_1 | BGC0001313 | Arabidiol\_/\_Baruol\_biosynthetic\_gene\_cluster. | 33 | 31.8811881188 | 80 | 5.6e-15 |
| NP\_199610.1 | cytochrome\_P450\_705A5 | BGC0000670 | Thalianol\_biosynthetic\_gene\_cluster | 29 | 34.2574257426 | 79 | 9.6e-15 |
| AEE83590.1 | cytochrome\_P450,\_family\_705,\_subfamily\_A,\_polypeptide\_4 | BGC0001313 | Arabidiol\_/\_Baruol\_biosynthetic\_gene\_cluster. | 29 | 36.0396039604 | 78 | 2.1e-14 |
| BAE71332.1 | cytochrome\_P450\_monooxygenase | BGC0000004 | Aflatoxin\_biosynthetic\_gene\_cluster | 28 | 34.2574257426 | 75 | 1.8e-13 |
| AAS90107.1 | CypX | BGC0000006 | Aflatoxin\_biosynthetic\_gene\_cluster | 28 | 34.2574257426 | 75 | 1.8e-13 |
| AAS90082.1 | CypX | BGC0000010 | Aflatoxin\_biosynthetic\_gene\_cluster | 28 | 34.2574257426 | 75 | 1.8e-13 |
| NP\_199072.1 | cytochrome\_P450,\_family\_705,\_subfamily\_A,\_polypeptide\_12 | BGC0000669 | Marneral\_biosynthetic\_gene\_cluster | 30 | 37.4257425743 | 74 | 3.1e-13 |
| AAS90014.1 | CypX | BGC0000007 | Aflatoxin\_biosynthetic\_gene\_cluster | 27 | 34.2574257426 | 73 | 9e-13 |
| AAS90062.1 | CypX | BGC0000009 | Aflatoxin\_biosynthetic\_gene\_cluster | 26 | 38.8118811881 | 72 | 1.5e-12 |
| AAS90036.1 | CypX | BGC0000008 | Aflatoxin\_biosynthetic\_gene\_cluster | 27 | 34.2574257426 | 72 | 1.5e-12 |
| AEE83588.1 | cytochrome\_P450,\_family\_705,\_subfamily\_A,\_polypeptide\_3 | BGC0001313 | Arabidiol\_/\_Baruol\_biosynthetic\_gene\_cluster. | 29 | 29.900990099 | 70 | 7.6e-12 |
| AAC49192.1 | putative\_p450\_monooxygenase | BGC0000152 | Sterigmatocystin\_biosynthetic\_gene\_cluster | 28 | 40.198019802 | 70 | 7.6e-12 |
| AFW60200.1 | benzoxazinone\_synthesis3 | BGC0000810 | Benzoxazinone\_DIMBOA\_biosynthetic\_gene\_cluster | 25 | 52.4752475248 | 69 | 9.9e-12 |
| ACH72911.1 | AflV | BGC0000011 | Aflatoxin/sterigmatocystin\_biosynthetic\_gene\_cluster | 26 | 34.2574257426 | 62 | 1.2e-09 |
| EER93095.1 | hypothetical\_protein | BGC0000798 | Dhurrin\_biosynthetic\_gene\_cluster | 25 | 35.2475247525 | 57 | 5.1e-08 |
| CYP719A21 |  | BGC0001325 | Noscapine\_biosynthetic\_gene\_cluster. | 27 | 31.8811881188 | 57 | 5.1e-08 |
